# Identification of an endoplasmic reticulum stress-associated gene signature to predict the immune status and prognosis of cutaneous melanoma

**DOI:** 10.1097/MD.0000000000030280

**Published:** 2022-09-09

**Authors:** Rong Chen, Linjun Niu, Liang Wu, Youwu He, Gang Liu, Kangjie Hong

**Affiliations:** a Department of Hand Plastic Surgery, The First People’s Hospital of Linping District, Hangzhou, China; b Department of Oncology, Huaibei People’s Hospital, Anhui, China; c Department of Neurology, Chun’an First People’s Hospital, Hangzhou, China.

**Keywords:** cutaneous melanoma, drug sensitivity, endoplasmic reticulum stress, gene signature, immune microenvironment, immune status, prognosis

## Abstract

Besides protecting normal cells from various internal and external perturbations, endoplasmic reticulum (ER) stress is also directly related to the pathogenesis of cutaneous melanoma (CM). However, due to the lack of specific molecular biomarkers, ER stress has not been considered a novel treatment target for CM. Here, we identified ER stress-related genes involved in the prognosis of CM patients and constructed an effective model for the prognostic prediction of these patients. First, gene expression data of CM and normal skin tissues from the Genotype-Tissue Expression (GTEx) and The Cancer Genome Atlas (TCGA) databases were retrieved to identify differentially expressed ER stress-related genes in CM. Meanwhile, an independent cohort obtained from the Gene Expression Omnibus (GEO) database was used for validation. The ER stress genes (*ZBP1*, *DIABLO*, *GNLY*, *FASLG*, *AURKA*, *TNFRSF21*, and *CD40LG*) that were associated with CM prognosis were incorporated into our prognostic model. The functional analyses indicated that the prognostic model was correlated with patient survival, gender, and cancer growth. Multivariate and univariate Cox regressions revealed that the constructed model could serve as an independent prognostic factor for CM patients. The pathway enrichment analysis showed that the risk model was enriched in different immunity and cancer progression-associated pathways. Moreover, the signature model was significantly connected with the immune subtypes, infiltration of immune cells, immune microenvironment, as well as tumor stem cells. The gene function analysis revealed that 7 ER stress genes were differentially expressed in CM patients and were significantly associated with prognosis and several antitumor drugs. Overall, our current model presented predictive value for the prognosis of CM patients and can be further used in the development of novel therapeutic strategies for CM.

## 1. Introduction

Cutaneous melanoma (CM) is an aggressive malignant tumor that threatens human life.^[1]^ The pathogenesis and development of CM are negatively correlated with skin pigmentation. Most cases comprise patients with low skin pigmentation and who were exposed to ultraviolet radiation.^[2]^ The production of melanin involves the synthesis and interaction of multiple proteins in melanosomes. It starts by transforming l-DOPA and l-tyrosine onto melanin polymers that can protect melanocytes from various physical and chemical threats.^[3–5]^ Through modulating aerobic glycolysis, oxygen consumption,^[6,7]^ intermediates of melanogenesis,^[8,9]^ and the interaction with several metabolic pathways,^[10]^ melanogenesis can also affect the behavior of malignant and normal melanocytes, and ultimately influence the outcome of melanoma treatments. In 2018, a total of 287,723 people were diagnosed with melanoma worldwide, and 60,709 died due to this disease.^[11]^ The 10-year overall survival (OS) rates of stages I and II CM patients are 75 and 98%, respectively.^[12]^ On the other hand, only 24 and 88% of CM patients in stages IIIA to IIID survived after 10 years, compared to those in stages I and II, suggesting that the early diagnosis of CM might affect its outcome. It has been suggested that skin pigmentation is also involved in tumorigenesis and progression, while the pathogenesis is affected by genetic susceptibility, family history, and acquired melanocytic nevi.^[13,14]^ However, the precise pathogenic mechanisms behind CM remain unknown. Hence, accurate diagnosis in relatively early stages can significantly influence CM therapies. Recently, many investigators have attempted to identify novel biomarkers that can be used for prognostic prediction and personalized therapy of CM patients, but only a few biomarkers of clinical significance were identified.^[15]^ Therefore, the identification of new biomarkers that can accurately predict the prognosis of CM patients is urgently needed.

The endoplasmic reticulum (ER) is a prominent organelle in eukaryotic cells and is involved in the folding and synthesis of transmembrane and secretory proteins, calcium homeostasis regulation, and lipid biosynthesis.^[16]^ During internal and external perturbations, the ER can be induced by several types of stress, such as the disruption of redox homeostasis, nutrient deprivation, and inflammatory stimuli.^[17]^ In skin tissues, the ER stress-induced unfolded protein response (UPR) is necessary for cell differentiation. However, chronic and continuous activation of UPR can ultimately develop into a cell death mechanism^[18]^ and result in some skin diseases, including melanoma.^[19]^ Recently, increasing evidence has shown that ER stress participates in melanoma malignancy and progression by interacting with GRP78/BiP, autophagy, and forkhead family transcription factor (FOXO) pathways.^[20–22]^ Moreover, ER stress has become a potential and prevalent target in cancer treatment.^[23]^ Although the connection between ER stress and melanoma is widely accepted, few specific genetic markers of ER stress have been identified in melanoma.

Through bioinformatic analysis, many disease-specific biomarkers have been identified. However, ER stress genes associated with melanoma progression or prognosis were not previously identified by systematic studies. Therefore, we conducted the analysis of differential gene expression and univariate Cox regression in this study, and identified the genes differentially expressed and correlated with the prognosis of CM patients. Then, hub ER stress-related genes were characterized, and a gene risk model was constructed using the least absolute shrinkage and selection operator (LASSO). The prognostic value and clinical significance of this model were further validated in CM patients. Moreover, we analyzed the connections between the ER stress-related gene signature and immune infiltrates, immune microenvironment, relationship with m6A genes, tumor stemness, and cancer chemoresistance. Currently, most models are generated according to tumor immunity and miRNAs, while a thorough analysis of ER stress genes in CM has not been performed yet. Thus, our present study demonstrated that ta risk signature with ER stress-related genes can be used to predict the prognosis of CM patients.

## 2. Materials and methods

### 2.1. Datasets

The RNA-sequencing data and related clinical information of CM patients (n = 471) and normal control (n = 1) were retrieved from the TCGA database (https://portal.gdc.cancer.gov) on June 30, 2020. Transcriptome data for 812 normal skin samples were downloaded from the GTEx database. Meanwhile, clinical information and gene profiles of CM patients (n = 214) were obtained from the GEO database (ID: GSE59455 and GSE65904) and used as a cohort for external validation. Additionally, to remove batch effects, the “sva” R package was used to perform log_2_-transformations and normalize the results.^[24,25]^ Furthermore, the protein domains of ER stress genes (n = 583) were retrieved from the human gene database—GeneCards (Supplementary Table 1, Supplementary Digital Content 1, http://links.lww.com/MD/H128).

### 2.2. Construction of the prognostic gene signature

A differential expression analysis using the “limma” package was conducted to identify the Differentially expressed ER stress genes (DEERGs). An FDR < 0.05 and | log_2_ fold change (FC)| > 1 were used to identify the candidate DEERGs. The Kaplan–Meier survival package and univariate Cox regression were employed to identify the prognostic genes associated with ER stress in the TCGA-CM cohort (a *P* < .05 was set as the cutoff). Overlapping genes between the candidate DEERGs and Cox regression analysis were selected and visualized in a Venn diagram. Then, to generate a risk signature for CM and identify hub ER stress-related genes, LASSO analysis was used to integrate the selected genes. The formula risk   score= Σ expgenei * β i was used to calculate the risk score for CM patients. In this formula, the relative expression of genes related to pyroptosis is represented as “expgenei” and the regression coefficients as i and β.^[26]^ Next, we divided all CM patients into 2 groups according to risk scores. The same regression coefficients and median risk scores were applied to stratify the patients in the validation cohort into 2 risk groups.

### 2.3. Evaluation of the risk signature

The “Ggplot2” and “Rtsne” R packages were applied to explore the distribution of the 2 risk groups in the constructed models by t-SNE and Principal Component Analysis (PCA). The “survival” R package was used to compare the outcomes between the 2 risk groups, according to their risk levels. The predictive accuracy and clinical characteristics of the risk signature were evaluated using the “timeROC” R package and Cox regression analyses. Moreover, the “ggpubr” R package was used to visualize the connections between clinical characteristics and the risk signature in the TCGA-CM cohort.

### 2.4. Kyoto encyclopedia of genes and genomes (KEGG) and gene ontology (GO) enrichment analyses

The Gene Set Enrichment Analysis (GSEA) software version 4.1 was used to compare the KEGG enrichment between the 2 risk groups. Meanwhile, the Integrated Discovery, Visualization, and Annotation database (version 6.8)^[27]^ was used to establish the biological function of ER stress genes based on GO and KEGG enrichment analyses. Statistical significance was considered when both FDR and *p* were < 0.05.

### 2.5. Tumor microenvironment (TME) and immune response analyses

The infiltration of stromal and immune cells into tumors was determined using stromal and immune scores.^[28]^ Furthermore, Spearman correlations were employed to examine the relationship between the score of risk and the scores of stromal and immune. Two-way ANOVA was used to identify the connections between risk scores and immune infiltration subtypes. Finally, the 2-way ANOVA analysis was carried out to identify the connections of risk scores and immune infiltration subtypes. To determine the gene signature’s stem cell-like features, Spearman correlations were conducted to evaluate the connections between tumor stemness and scores of signature.

### 2.6. Chemotherapy sensitivity analysis

First, 218 chemotherapy drugs (Supplementary Table 2, Supplementary Digital Content 2, http://links.lww.com/MD/H129) were retrieved from the CellMiner database (https://discover.nci.nih.gov/cellminer), after filtering standard FDA certifications and clinical laboratory verifications. Then, Pearson correlation analyses were performed to determine the sensitivity of hub ER stress genes to chemotherapy drugs. The “ggplot2”, “limma”, “ggpubr”, and “impute” R packages were employed to visualize these results.

## 3. Results

### 3.1. Screening of prognosis-associated DEERGs

**Figure 1.**
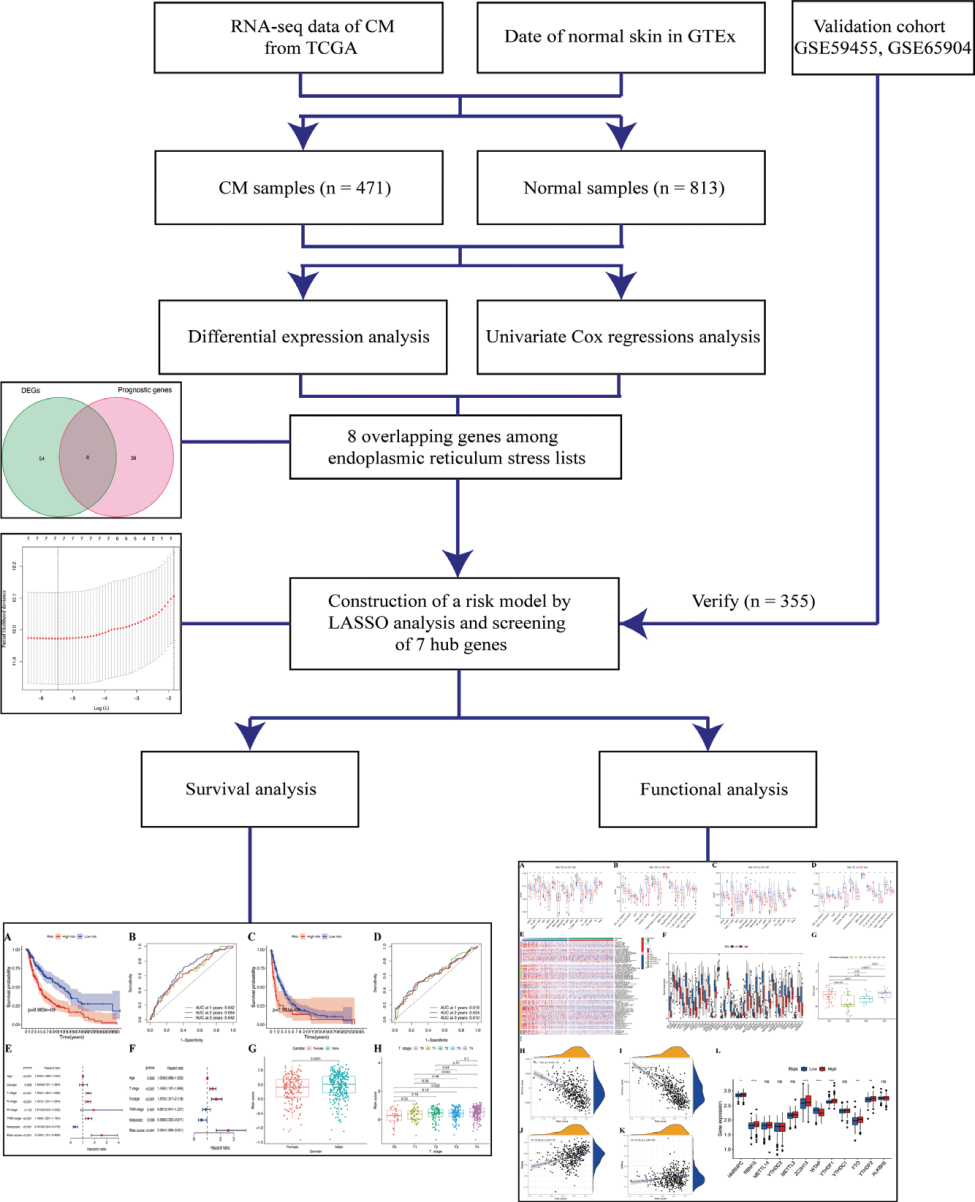
Schema of the study.

**Figure 2.**
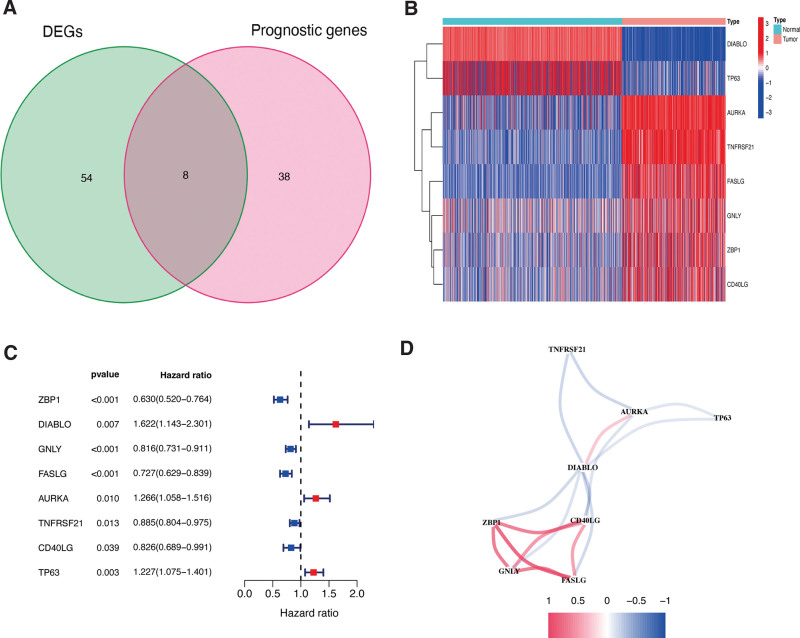
Identification of candidate prognostic DEGs in TCGA-CM cohort. (A) Venn diagram of ER-related genes determined by differential expression and univariate Cox analyses. (B) Heatmap of candidate prognostic DEGs in TCGA-CM cohort. (C) Forest plots of correlations between candidate prognostic DEGs and overall survival of patients in TCGA-CM cohort. (D) Correlation network of candidate prognostic DEGs.

The datasets analyzed in the present study are presented in Figure 1. A total of 583 ER stress genes were selected from 813 normal and 471 CM tissues, then analyzed to identify DEERGs. Sixty-two ER stress genes were differentially expressed in the TCGA dataset. Among them, 8 were connected with the OS of CM patients (Fig. 2A). The distribution of these genes in normal and tumor samples is shown in Figure 2B. Meanwhile, their association with prognosis was verified using univariate Cox analysis (Fig. 2C). These associations were also observed using correlations (Fig. 2D). Finally, 8 overlapping ER stress genes were identified.

### 3.2. Construction of a genetic score model for CM patients

**Figure 3.**
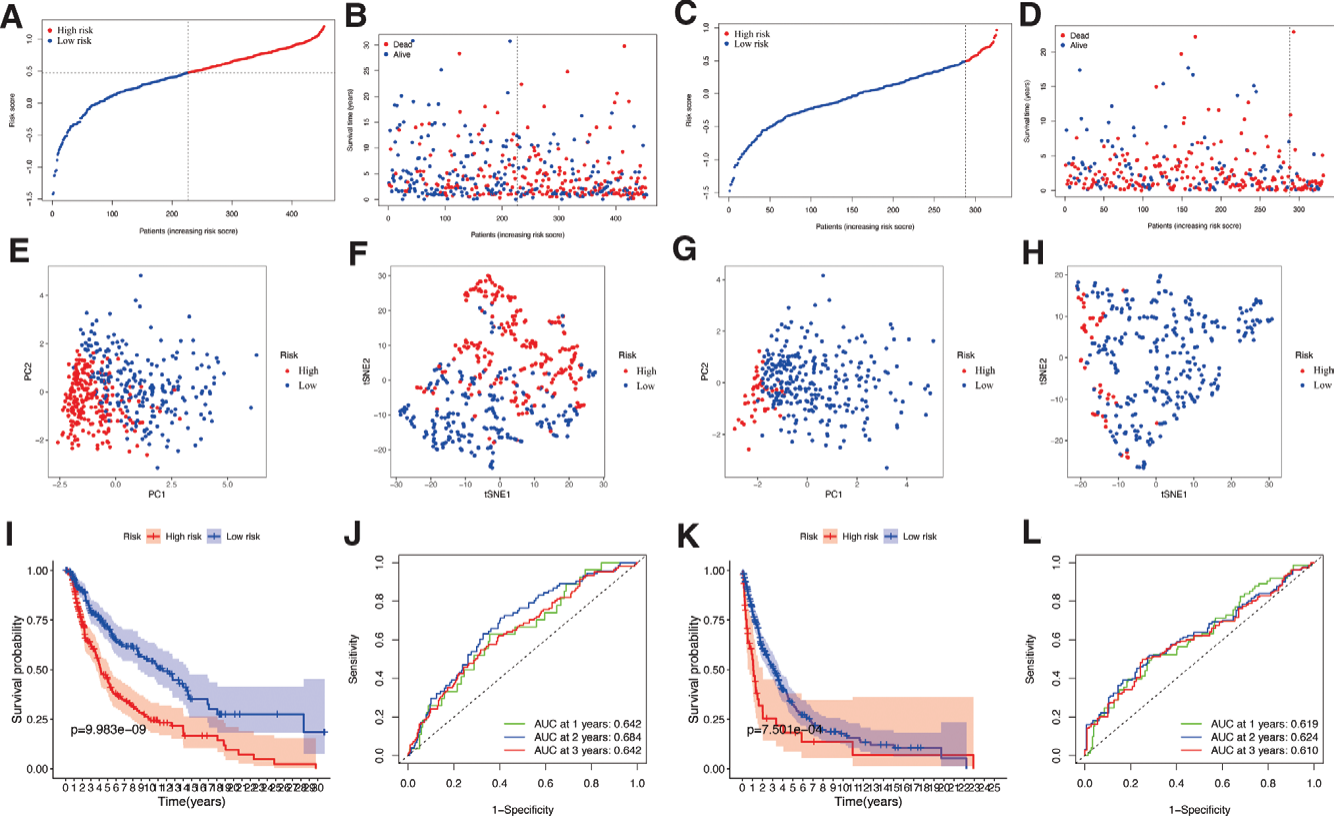
Prognostic analysis of risk signature. Risk score distribution (A, C) and survival status (B, D) of TCGA-CM and GEO cohorts, respectively. PCA plot (E) and t-SNE (F) analysis of TCGA-CM cohort. PCA plot (G) and t-SNE (H) analysis of validation cohort. (I) Survival curve of the TCGA cohort. (J) TimeROC curves to forecast overall survival of patients from TCGA-CM cohort. (K) Survival curve of validation cohort. (L) TimeROC curves to forecast overall survival of patients in validation cohort.

Further, the 8 DEERGs identified were analyzed using LASSO. Seven ER stress genes were selected to construct the risk signature model: Z-DNA Binding Protein 1 (ZBP1), Diablo IAP-Binding Mitochondrial Protein (DIABLO), Granulysin (GNLY), Fas Ligand (FASLG), Aurora Kinase A (AURKA), TNF Receptor Superfamily Member 21 (TNFRSF21), and CD40 Ligand (CD40LG) (Table 1, Supplementary Figure 1, Supplementary Digital Content 3, http://links.lww.com/MD/H130). According to the median risk scores, the patients in TCGA (Fig. 3A and B) and GEO (Fig. 3C and D) cohorts were divided into high- and low-risk groups. The different directions distributed in the 2 groups were found in both TCGA (Fig. 3E and F) and GEO (Fig. 3G and H) cohorts during the PCA and t-SNE.

### 3.3. Correlations between the risk scores and clinical characteristics of CM patients

In the TCGA cohort, reduced OS was observed for high-risk CM patients (*P* < .001; Fig. 3I), further confirmed in the validation cohort (*P* < .001; Fig. 3K). The Receiver Operating Characteristic (ROC) analysis indicated that our risk signature presented a moderate predictive accuracy at 1 (AUC = 0.642), 2 (AUC = 0.684), and 3 (AUC = 0.642) years of follow-up in the TCGA-CM cohort (Fig. 3J). Compared with the TCGA cohort, the AUC slightly decreased in the validation cohort, presenting an AUC of 0.619 for 1, 0.624 for 2, and 0.610 for 3 years of follow-up (Fig. 3L). These results confirmed that our signature model could sensitively and specifically predict the OS of CM patients.

**Figure 4.**
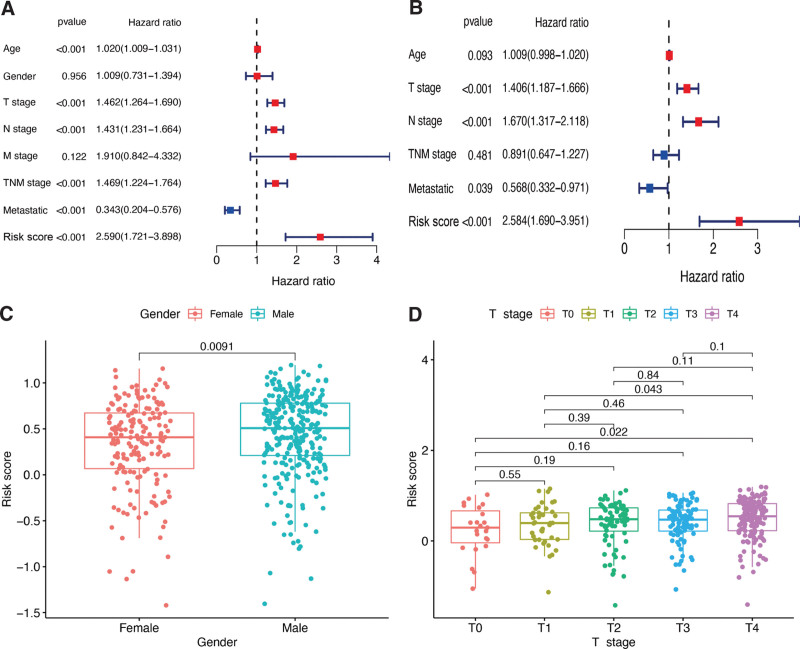
Associations between risk signature and clinicopathological factors. Univariate (A) and multivariate Cox (B) regression of clinicopathological features in TCGA-CM cohort. Correlations between risk scores and gender (C) and T stage (D) in TCGA-CM cohort.

Multivariate and univariate Cox regressions showed that, in CM patients, the risk score was an independent prognostic factor, whereas the risk signature was associated with prognosis (Fig. 4A and B). Moreover, higher risk scores were observed in male CM patients (*P* < .05, Fig. 4C). Meanwhile, a significant correlation between higher T stages and higher risk scores was observed in CM patients (*P* < .05, Fig. 4D). These analyses indicated that our risk signature is connected to CM development.

### 3.4. Correlations with immune response, tumor stemness, and m6a-related genes

**Figure 5.**
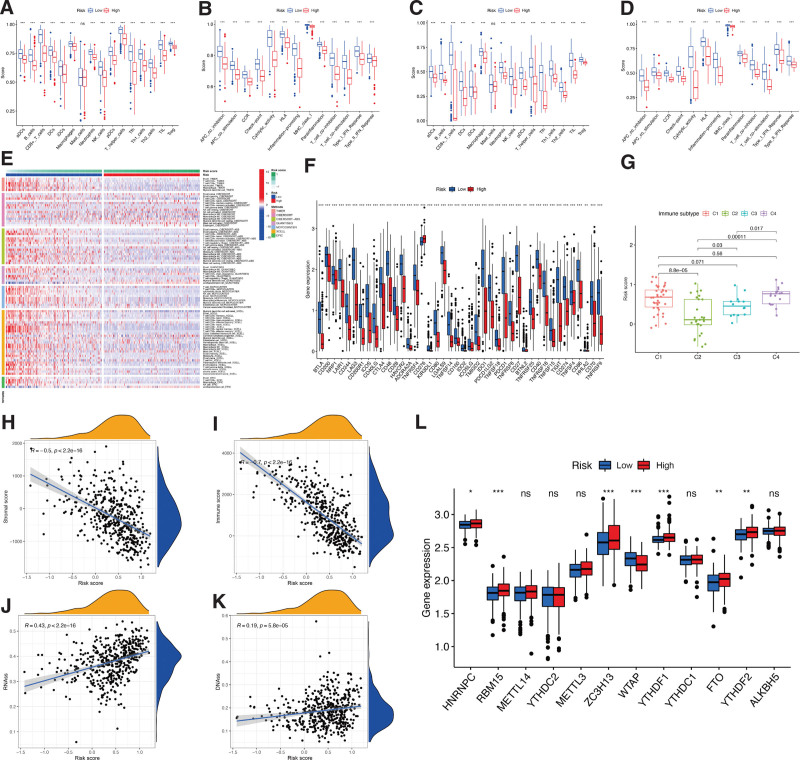
Potential role of risk signature in CM immune status, tumor stemness, and m6A-related genes. Boxplots of scores of immune cells (A) and immune-associated functions (B) in risk subgroups of TCGA-CM cohort. Boxplots of scores for immune cells (C) and immune-associated functions (D) in risk subgroups of validation cohort. (E) Heatmap for immune responses based on EPIC, XCELL, MCP counter, QUANTISEQ, CIBERSORT, and TIMER among 2 risk subgroups. Associations between risk signature and immune checkpoints (F), immune infiltration subtypes (G), stromal scores (H), immune scores (I), RNAss (J), DNAss (K), and m6A-related genes (L).

Compared to the low-risk score subgroup, the levels of nearly all related functions, pathways, and the proportion of immune cell subpopulations (except mast cells) were greatly reduced in the high-risk score group, in both TCGA (Fig. 5A and B) and validation (Fig. 5C and D) cohorts. Similar results were obtained using EPIC, MCP counter, XCELL, CIBERSORT, QUANTISEQ, and TIMER (Fig. 5C). Regarding immune checkpoints, all ER stress-related genes identified were more expressed in the low-risk subgroup, except for *CD276* (Fig. 5F). Moreover, for tumor promotion and suppression, the immune infiltrates, including wound healing (C1), INF-g dominant (C2), inflammatory (C3), and lymphocyte-depleted (C4), were calculated to explore the relationship between the risk signature and immune components.^[29]^ The results showed that the risk scores were significantly higher for C1 and C4 subtypes and lower for C2 (Fig. 5G). Meanwhile, considering the connection between the ER stress genes identified and immune components, the genes *ZBP1*, *GNLY*, *FASLG*, and *CD40LG* were upregulated (Supplementary Figure 2A–D, Supplementary Digital Content 4, http://links.lww.com/MD/H131) and significantly connected with the C2 immune subtype. On the other hand, C1 and C4 subtypes were connected to the downregulation of these genes. Meanwhile, no significant differences were detected between immune subtypes and the risk scores of *DIABLO*, *AURKA*, and *TNFRSF21* genes (Supplementary Figure 2E–G, Supplementary Digital Content 4, http://links.lww.com/MD/H131).

Other possible regulators of CM progression, such as tumor immune microenvironment (stromal and immune scores), m6A-related genes, as well as tumor stemness (DNA methylation pattern and RNA stemness score). The gene signature constructed was significantly and negatively associated with the immune microenvironment (*P* < .05; Fig. 5H and I), but positively associated with tumor stemness (*P* < .05; Fig. 5J and K). The correlation between hub ER stress genes and tumor immune microenvironment and stemness (Supplementary Figure 3, Supplementary Digital Content 5, http://links.lww.com/MD/H132) showed that *ZBP1*, *GNLY*, *FASLG*, *TNFRSF21*, and *CD40LG* were positively connected but *DIABLO* and *AURKA* were negatively associated with the tumor immune microenvironment; meanwhile, *ZBP1*, *FASLG*, *TNFRSF21*, and *CD40LG* were negatively associated but *DIABLO* and *AURKA* were positively related with tumor stemness. Besides, no significant association was observed between *GNLY* and DNAss and RNAss. The m6A-related genes *HNRNPC, RBM15*, ZC3H13, *YTHDF1*, *YTHDF2*, and *FTO* were more expressed and *WTAP* was less expressed in the high-risk group in comparison to the low-risk group (Figure 5L).

**Figure 6.**
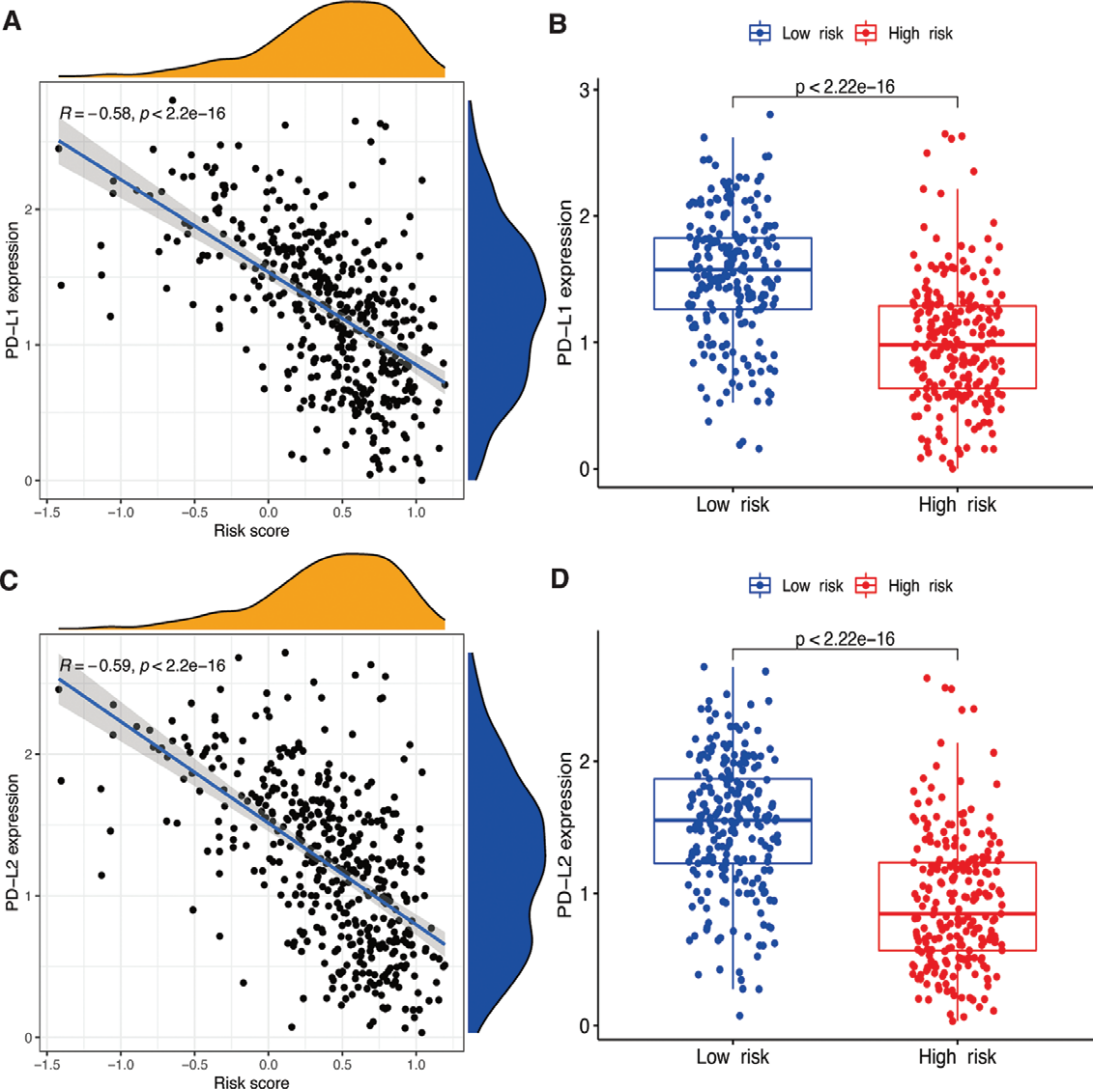
Associations between risk signature and immune checkpoints. Expression levels of genes PD-L1 (A) and PD-L2 (C) in risk subgroups. Correlation analysis between risk score, PD-L1 (B), and PD-L2 (D).

Moreover, considering the roles of the immune checkpoint proteins PD-L1 and PD-L2 in immune progression, we analyzed the correlation between these loci and the gene signature. Both *PD-L1* and *PD-L2* were significantly less expressed in the high-risk group and were negatively related to the CM risk signature (Figure 6A–D).

### 3.5. Functional enrichment analyses

**Figure 7.**
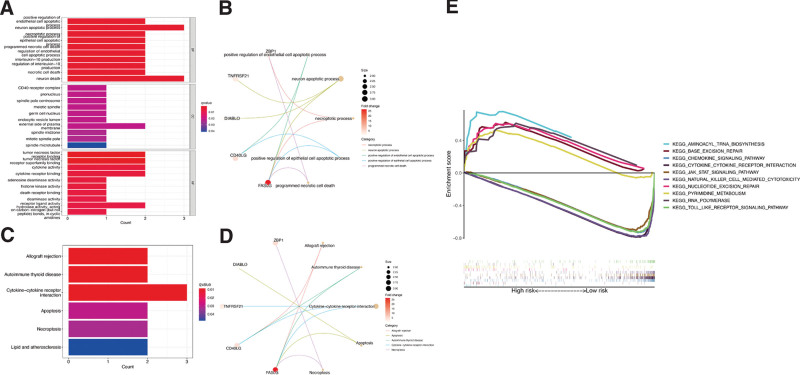
Functional enrichment analysis. (A, B) GO enrichment terms of hub ER genes in CC, BP, and MF. (C, D) KEGG enrichment terms of hub ER genes. (E) Gene Set Enrichment Analysis of top 10 enriched pathways in risk signature.

In the GO analysis, predominant enrichment of hub genes was observed in different biological processes (BP), including neuron apoptotic process, positive regulation of endothelial cell apoptotic process, and necroptotic process (Figure 7A and B). In the cellular components (CC) category, hub ER stress-related genes were enriched in the CD40 receptor complex, pronucleus, and spindle pole centrosome. Moreover, tumor necrosis factor receptor binding, cytokine activity, and cytokine receptor binding were enriched in the molecular functions (MF) category. Meanwhile, the KEGG enrichment analysis indicated that the genes associated with pyroptosis were enriched in the allograft rejection, autoimmune thyroid disease, cytokine-cytokine receptor interaction, apoptosis, and necroptosis pathways (Figure 7C and D). Additionally, the KEGG enrichment analysis carried out using the GSEA software (Figure 7E) revealed that, in the high-risk group, 19 pathways, including pyrimidine metabolism, nucleotide excision repair, and RNA polymerase, were significantly enriched (Supplementary Table 3, Supplementary Digital Content 6, http://links.lww.com/MD/H133). Meanwhile, in the group with low-risk scores, 46 pathways, including chemokine signaling pathways, Jak stat signaling pathway, and natural killer cell-mediated cytotoxicity, were enriched. Altogether, these results indicated possible underlying mechanisms of ER stress genes in CM.

### 3.6. Prognostic value of the hub genes selected

**Figure 8.**
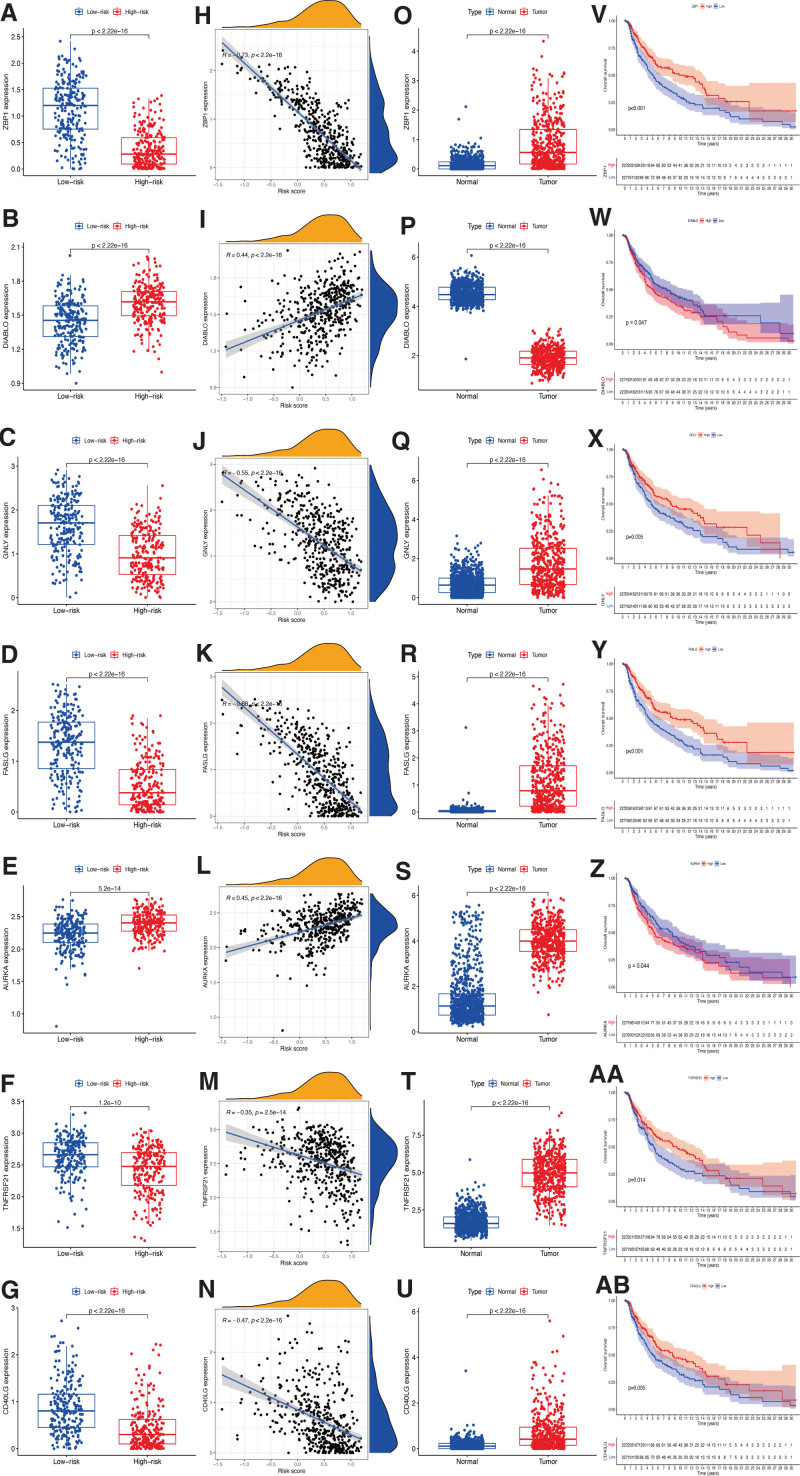
Roles of ER stress genes in risk signature and CM prognosis. Expression of *ZBP1* (A), *DIABLO* (B), *GNLY* (C), *FASLG* (D), *AURKA* (E), *TNFRSF21* (F), and *CD40LG* (G) genes in risk subgroups. Correlations between risk signature and *ZBP1* (H), *DIABLO* (I), *GNLY* (J), *FASLG* (K), *AURKA* (L), *TNFRSF21* (M), and *CD40LG* (N) genes. Expression of *ZBP1* (O), *DIABLO* (P), *GNLY* (Q), *FASLG* (R), *AURKA* (S), *TNFRSF21* (T), and *CD40LG* (U) genes in CM. Kaplan–Meier curves of TCGA-CM cohort verify prognostic value of *ZBP1* (V), *DIABLO* (W), *GNLY* (X), *FASLG* (Y), *AURKA* (Z), *TNFRSF21* (AA), and *CD40LG* (AB).

In the high-risk group, significantly elevated gene expressions were found for the ER stress genes *DIABLO* and *AURKA* compared to the low-risk group. Meanwhile, ZBP1, GNLY, FASLG, TNFRSF21, and CD40LG were less expressed (Figure 8A–G). Then, the correlation analyses showed that the expression of *DIABLO* and *AURKA* was positively associated, and ZBP1, GNLY, FASLG, TNFRSF21, and CD40LG were negatively associated with risk scores (Figure 8H–N). Considering the correlation of ER stress genes and CM tissues, abundant expression of ZBP1, GNLY, FASLG, AURKA, TNFRSF21, and CD40LG, and less *DIABLO* expression were observed in CM samples than in healthy controls (Figure 8O–U). Finally, the Kaplan–Meier survival analysis was used to examine the prognostic value of ER stress genes. the expression of *DIABLO* and *AURKA* was negatively associated with the prognosis of CM patients, while ZBP1, GNLY, FASLG, TNFRSF21, and CD40LG were positively associated with survival (Figure 8V–AB).

### 3.7. Connections between hub ER stress genes and drug sensitivity

**Figure 9.**
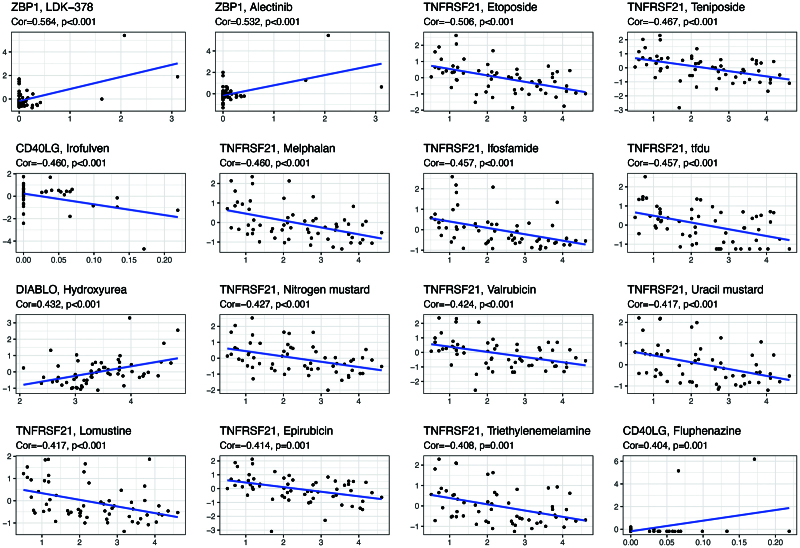
Scatter plots of top 16 classes of associations between ER stress genes and drug sensitivity.

Hub ER stress genes were sensitively and respectively correlated to chemotherapy drugs (*P* < .05; Supplementary Table 4, Supplementary Digital Content 7, http://links.lww.com/MD/H134). For example, the increased sensitivity to LDK-378 and alectinib was positively correlated with increased ZBP1 expression (**Fig. 9**). In contrast, increased TNFRSF21 expression was negatively associated with the sensitivity to etoposide, teniposide, melphalan, ifosfamide, tfdu, nitrogen mustard, valrubicin, uracil mustard, lomustine, epirubicin, and triethylenemelamine.

## 4. Discussion

Although increasing melanoma biomarkers have been identified using next-generation sequencing, novel markers associated with early prognosis and more close detection are still needed for CM patients. The ER stress is significantly correlated with skin cells differentiation, and can also participate in melanoma progression. Meanwhile, the role and function of ER stress in CM have not been studied systemically. Moreover, no ER stress-related gene signature has been identified yet. Similar to previously established risk signatures, such as immune checkpoint-,^[30]^ ferroptosis-,^[31]^ and hypoxia-related signatures,^[32]^ our current risk gene signature displayed high predictive accuracy for the prognosis of CM patients. This gene signature was also connected with the TME, immune components, immune status, tumor stemness, m6A-related genes, and susceptibility to chemotherapeutic drugs, thereby presenting an advantage over other gene signatures.

In the present study, 583 ER stress genes were systematically analyzed to identify those associated with the OS of CM patients. Next, 7 hub genes (ZBP1, DIABLO, GNLY, FASLG, AURKA, TNFRSF21, and CD40LG) were used to construct a novel prognostic signature for CM. The survival and ROC analyses indicated that the gene signature was not only significantly connected with the OS of CM patients but also showed high accuracy for prognosis prediction. The signature was also correlated with the T stage of CM, revealing the effectiveness of this gene signature for predicting the tumor growth of CM.

Based on the GSEA, the risk signature was enriched in several immune-related pathways, including the T cell receptor signaling pathway, the Toll-like receptor signaling pathway, as well as natural killer cell-mediated cytotoxicity. Thus, the prognostic value of the gene signature might be attributed to its association with immune processes. Nearly all immune cells, except mast cells, presented reduced infiltration and immune functions in the high-risk score group. Since these infiltrated immune cells play important roles in the stimulation of antitumor immunity,^[33]^ the degree of antitumor immunity of SKCM patients in the high-risk group was substantially reduced. Additionally, negative correlations between both of the immune and stromal cell scores and risk scores were observed from the ESTIMATE algorithm analysis, suggesting the inhibited infiltration of immune cells in the high-risk group. Moreover, regarding the correlation of CM and immune components, we found that C2 was significantly associated with the risk scores. Considering the predictive value of the gene signature in CM prognosis, C2 might also be a protective factor in CM.

Cancer immunotherapies targeting immune checkpoints have improved the outcomes of various cancers.^[34]^ However, they have different effects depending on the tumor type. Both PD-L1 and PD-L2 are virtual regulators of immune responses.^[35]^ Additionally, some tumors express immune-inhibitory checkpoint cytokines, contributing to the suppression of immune responses mediated by T cells. The binding of PD1 on T cells and its ligand PD-L1 in tumor cells can induce the immune escape of tumor cells and exhaustion of T cells.^[36]^ However, better clinical outcomes were also positively correlated with the expression level of PD-L1 on melanoma cells. Through blocking the PD1/PD-L1 binding-mediated inhibition and enhancing the function of T cells, impressive outcomes were observed after treatment with monoclonal antibodies targeting the PD-1/PD-L1 pathway in clinical trials.^[37,38]^ The significantly differential expression of PD-L1 and PD-L2 in our gene risk groups, as well as the fact that they are both negatively correlated with the risk scores, were also verified in this study. The levels of nearly all immune checkpoints were significantly lower in the high-risk group, suggesting that immune responses were greatly altered in this group. Overall, our prognostic gene signature could predict the expression of immune checkpoints in CM and potentially guide immunotherapy implementation. However, the specific relationship between ER stress genes and immune genes requires further study.

Cancer stem cell-like cells (CSCs) promote cancer progression due to their invasion and self-renewal abilities.^[39,40]^ In the present study, the ER stress gene signature was positively connected with stem cell score, confirming that this signature was a risk factor for CM. The m6A-related genes comprehend another tumor research hotspot.^[41]^ Our ER stress gene signature could effectively predict the expression levels of m6A-related genes in CM, including HNRNPC, RBM15, ZC3H13, WTAP, YTHDF1, FTO, and YTHDF2. However, the specific mechanisms underlying these relationships need further exploration.

Despite the prognostic value of the current risk signature, this study also has some limitations. First, the results from our present retrospective study need further confirmation by prospective studies. Second, more experimental assays are needed to verify and validate the conclusions obtained from bioinformatics analyses. In the future, functional studies should be performed to gain mechanistic insights into ER stress genes and their role in CM development.

## 5. Conclusions

In the present study, a novel prognostic risk signature consisting of 7 hub ER stress-related genes was constructed and presented high predictive accuracy. This gene signature was valuable to predict parameters related to immune components, immune functions, immune cell infiltration, tumor microenvironment, stemness, m6A-related genes, and drug sensitivity in CM patients. To the best of our knowledge, this is the first ER stress-associated gene signature for CM. These results also provided a novel basis for understanding the specific effects of ER stress genes in CM. Therefore, this study comprehends a significant contribution to the literature and can contribute to improvements in the outcomes and individualized treatments for CM patients

**Table 1:** Seven prognosis-associated endoplasmic reticulum stress genes in the TCGA-CM cohort were identified by LASSO analysis.

Gene name	Univariate Cox regression analysis		Differential gene expression analysis		LASSO coefficient
	HR	*P* value	LogFC	*P* value	
ZBP1	0.6300	2.62E-06	2.3428	2.89E-72	-0.5317
DIABLO	1.6218	0.0067	-1.2500	6.56E-196	0.1578
GNLY	0.8164	0.0003	1.3210	8.51E-55	-0.0618
FASLG	0.7266	1.32E-05	4.7369	1.03E-153	-0.0111
AURKA	1.2664	0.0102	1.4060	3.62E-145	0.2144
TNFRSF21	0.8854	0.0130	1.5088	1.69E-182	-0.0799
CD40LG	0.8263	0.0394	2.2427	1.01E-67	0.2784

## Author contributions

Conceptualization: Rong Chen.

Data curation: Rong Chen.

Formal analysis: Rong Chen.

Methodology: Linjun Niu.

Project administration: Kangjie Hong.

Writing—original draft: Liang Wu and Youwu He.

Writing—review & editing: Gang Liu

## Supplementary Material


