# Supplementary figures and images for: Identification of an endoplasmic reticulum stress-associated gene signature to predict the immune status and prognosis of cutaneous melanoma
Source: Medicine (Baltimore). 2022 Sep 9;101(36):e30280. doi: 10.1097/MD.0000000000030280 (PMC10980369; doi:10.1097/MD.0000000000030280)

**A**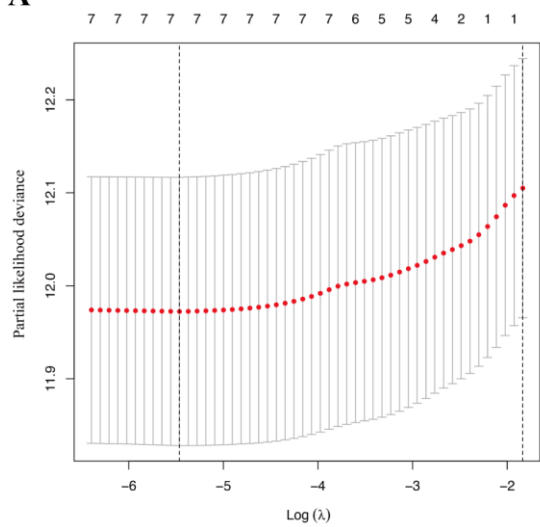**B**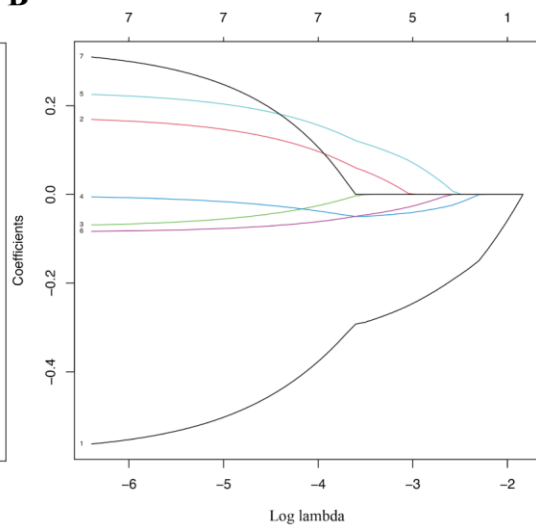

Supplement: Supplementary file 3 [file medi-101-e30280-s003.pdf]

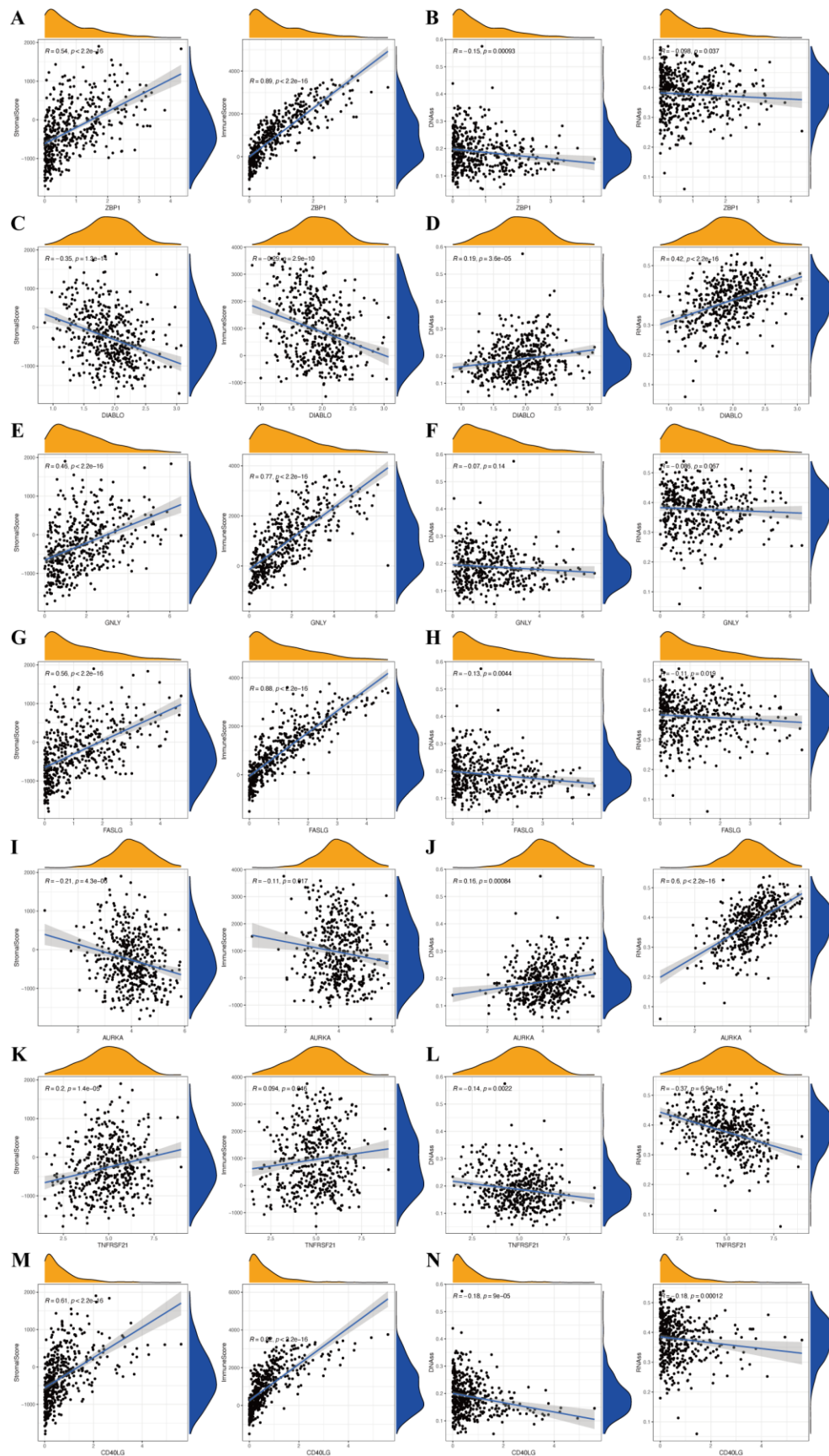

Supplement: Supplementary file 5 [file medi-101-e30280-s005.pdf]
