# Supplementary material for: Identification of an endoplasmic reticulum stress-associated gene signature to predict the immune status and prognosis of cutaneous melanoma
Source: Medicine (Baltimore). 2022 Sep 9;101(36):e30280. doi: 10.1097/MD.0000000000030280 (PMC10980369; doi:10.1097/MD.0000000000030280)
Supplement: Supplementary file 4 [file medi-101-e30280-s004.pdf]

Identification of an endoplasmic reticulum stress-associated gene signature to predict the immune status and prognosis of cutaneous melanoma

Running title: ER stress-associated signature of melanoma

Rong Chen[1], Linjun Niu[2], Liang Wu[1], Youwu He[1], Gang Liu[1], Kangjie Hong, MD, PhD[3*]

1 Department of Hand Plastic Surgery, The First People's Hospital of Linping District, Hangzhou, China, 311199

2 Department of Oncology，Huaibei People's Hospital, Anhui, China, 235099

3 Department of Neurology, Chun'an First People's Hospital, Hangzhou, China, 311700

Rong Chen and Linjun Niu contributed equally to this manuscript.

* Co-Corresponding authors.

Kangjie Hong: Tel: +86-19357325624. Fax: +86-07713131152. Academic/Institutional email address: kangjiehong123456@163.com

Abstract

Besides protecting normal cells from various internal and external perturbations, endoplasmic reticulum (ER) stress is also directly related to the pathogenesis of cutaneous melanoma (CM). However, due to the lack of specific molecular biomarkers, ER stress has not been considered a novel treatment target for CM. Here, we identified ER stress-related genes involved in the prognosis of CM patients and constructed an effective model for the prognostic prediction of these patients. First, gene expression data of CM and normal skin tissues from the Genotype-Tissue Expression (GTEx) and The Cancer Genome Atlas (TCGA) databases were retrieved to identify differentially expressed ER stress-related genes in CM. Meanwhile, an independent cohort obtained from the Gene Expression Omnibus (GEO) database was used for validation. The ER stress genes (*ZBP1*, *DIABLO*, *GNLY*, *FASLG*, *AURKA*, *TNFRSF21*, and *CD40LG*) that were associated with CM prognosis were incorporated into our prognostic model. The functional analyses indicated that the prognostic model was correlated with patient survival, gender, and cancer growth. Multivariate and univariate Cox regressions revealed that the constructed model could serve as an independent prognostic factor for CM patients. The pathway enrichment analysis showed that the risk model was enriched in different immunity and cancer progression-associated pathways. Moreover, the signature model was significantly connected with the immune subtypes, infiltration of immune cells, immune microenvironment, as well as tumor stem cells. The gene function analysis revealed that seven ER stress genes were differentially expressed in CM patients and were significantly associated with prognosis and several antitumor drugs. Overall, our current model presented predictive value for the prognosis of CM patients and can be further used in the development of novel therapeutic strategies for CM.

Abbreviations: CM = Cutaneous melanoma, ER = endoplasmic reticulum, TCGA = The Cancer Genome Atlas, GTEx = Genotype-Tissue Expression, GEO = Gene Expression Omnibus, GSEA = Gene Set Enrichment Analysis, OS = overall survival, DEGs = differential expressed genes, DEERGs = differentially expressed ER stress

genes, FC = fold change, GSEA = gene set enrichment analysis, LASSO = least absolute shrinkage and selection operator, GO = Gene Ontology, KEGG = Kyoto Encyclopedia of Genes and Genomes, BP = biological process, CC = cellular component, MF = molecular function, ROC = receiver operating characteristic, UPR = unfolded protein response, FOXO = forkhead family transcription factor, TME = tumor microenvironment, ZBP1 = Z-DNA Binding Protein 1, DIABLO = Diablo IAP-Binding Mitochondrial Protein, GNLY = Granulysin, FASLG = Fas Ligand, AURKA = Aurora Kinase A, TNFRSF21 = TNF Receptor Superfamily Member 21, CD40LG = CD40 Ligand, CSCs = cancer stem cell-like cells.

Keywords: Endoplasmic reticulum stress, prognosis, cutaneous melanoma, gene signature, immune microenvironment, immune status, drug sensitivity.

1. Introduction

Cutaneous melanoma (CM) is an aggressive malignant tumor that threatens human life [1]. The pathogenesis and development of CM are negatively correlated with skin pigmentation. Most cases comprise patients with low skin pigmentation and who were exposed to ultraviolet radiation [2]. The production of melanin involves the synthesis and interaction of multiple proteins in melanosomes. It starts by transforming L-DOPA and L-tyrosine onto melanin polymers that can protect melanocytes from various physical and chemical threats [3-5]. Through modulating aerobic glycolysis, oxygen consumption [6,7], intermediates of melanogenesis [8,9], and the interaction with several metabolic pathways [10], melanogenesis can also affect the behavior of malignant and normal melanocytes, and ultimately influence the outcome of melanoma treatments. In 2018, a total of 287,723 people were diagnosed with melanoma worldwide, and 60,709 died due to this disease [11]. The 10-year overall survival (OS) rates of stages I and II CM patients are 75 and 98%, respectively [12]. On the other hand, only 24 and 88% of CM patients in stages IIIA to IIID survived after 10 years, compared to those in stages I and II, suggesting that the early diagnosis of CM might affect its outcome. It has been suggested that skin pigmentation is also involved in tumorigenesis and progression, while the pathogenesis is affected by genetic susceptibility, family history, and acquired melanocytic nevi [13,14]. However, the precise pathogenic mechanisms behind CM remain unknown. Hence, accurate diagnosis in relatively early stages can significantly influence CM therapies. Recently, many investigators have attempted to identify novel biomarkers that can be used for prognostic prediction and personalized therapy of CM patients, but only a few biomarkers of clinical significance were identified [15]. Therefore, the identification of new biomarkers that can accurately predict the prognosis of CM patients is urgently needed.

The endoplasmic reticulum (ER) is a prominent organelle in eukaryotic cells and is involved in the folding and synthesis of transmembrane and secretory proteins, calcium homeostasis regulation, and lipid biosynthesis [16]. During internal and external

perturbations, the ER can be induced by several types of stress, such as the disruption of redox homeostasis, nutrient deprivation, and inflammatory stimuli [17]. In skin tissues, the ER stress-induced unfolded protein response (UPR) is necessary for cell differentiation. However, chronic and continuous activation of UPR can ultimately develop into a cell death mechanism [18] and result in some skin diseases, including melanoma [19]. Recently, increasing evidence has shown that ER stress participates in melanoma malignancy and progression by interacting with GRP78/BiP, autophagy, and forkhead family transcription factor (FOXO) pathways [20-22]. Moreover, ER stress has become a potential and prevalent target in cancer treatment [23]. Although the connection between ER stress and melanoma is widely accepted, few specific genetic markers of ER stress have been identified in melanoma.

Through bioinformatic analysis, many disease-specific biomarkers have been identified. However, ER stress genes associated with melanoma progression or prognosis were not previously identified by systematic studies. Therefore, we conducted the analysis of differential gene expression and univariate Cox regression in this study, and identified the genes differentially expressed and correlated with the prognosis of CM patients. Then, hub ER stress-related genes were characterized, and a gene risk model was constructed using the least absolute shrinkage and selection operator (LASSO). The prognostic value and clinical significance of this model were further validated in CM patients. Moreover, we analyzed the connections between the ER stress-related gene signature and immune infiltrates, immune microenvironment, relationship with m6A genes, tumor stemness, and cancer chemoresistance. Currently, most models are generated according to tumor immunity and miRNAs, while a thorough analysis of ER stress genes in CM has not been performed yet. Thus, our present study demonstrated that ta risk signature with ER stress-related genes can be used to predict the prognosis of CM patients.

2. Materials and Methods

2.1.Datasets

The RNA-sequencing data and related clinical information of CM patients (n = 471) and normal control (n = 1) were retrieved from the TCGA database (https://portal.gdc.cancer.gov) on June 30, 2020. Transcriptome data for 812 normal skin samples were downloaded from the GTEx database. Meanwhile, clinical information and gene profiles of CM patients (n = 214) were obtained from the GEO database (ID: GSE59455 and GSE65904) and used as a cohort for external validation. Additionally, to remove batch effects, the “sva” R package was used to perform $\log_2$-transformations and normalize the results [24,25]. Furthermore, the protein domains of ER stress genes (n = 583) were retrieved from the human gene database – GeneCards (**Supplementary Table 1,Supplementary Digital Content 1, http://links.lww.com/MD/H128**).

2.2.Construction of the prognostic gene signature

A differential expression analysis using the “limma” package was conducted to identify the Differentially expressed ER stress genes (DEERGs). An FDR < 0.05 and $|\log_2$ fold change (FC)$| > 1$ were used to identify the candidate DEERGs. The Kaplan-Meier survival package and univariate Cox regression were employed to identify the prognostic genes associated with ER stress in the TCGA-CM cohort (a $p < 0.05$ was set as the cutoff). Overlapping genes between the candidate DEERGs and Cox regression analysis were selected and visualized in a Venn diagram. Then, to generate a risk signature for CM and identify hub ER stress-related genes, LASSO analysis was used to integrate the selected genes. The formula $\text{risk score} = \Sigma \text{expgenei} * \beta\text{i}$ was used to calculate the risk score for CM patients. In this formula, the relative expression of genes related to pyroptosis is represented as “expgenei” and the regression coefficients as i and β [26]. Next, we divided all CM patients into two groups according to risk scores. The same regression coefficients and median risk scores were applied to stratify the patients in the validation cohort into two risk groups.

2.3.Evaluation of the risk signature

The “Ggplot2" and “Rtsne” R packages were applied to explore the distribution of the two risk groups in the constructed models by t-SNE and Principal Component Analysis (PCA). The "survival” R package was used to compare the outcomes between the two risk groups, according to their risk levels. The predictive accuracy and clinical characteristics of the risk signature were evaluated using the “timeROC” R package and Cox regression analyses. Moreover, the “ggpubr” R package was used to visualize the connections between clinical characteristics and the risk signature in the TCGA-CM cohort.

2.4.Kyoto Encyclopedia of Genes and Genomes (KEGG) and Gene Ontology (GO) enrichment analyses

The Gene Set Enrichment Analysis (GSEA) software version 4.1 was used to compare the KEGG enrichment between the two risk groups. Meanwhile, the Integrated Discovery, Visualization, and Annotation database (version 6.8) [27] was used to establish the biological function of ER stress genes based on GO and KEGG enrichment analyses. Statistical significance was considered when both FDR and p were < 0.05.

2.5.Tumor Microenvironment (TME) and immune response analyses

The infiltration of stromal and immune cells into tumors was determined using stromal and immune scores [28]. Furthermore, Spearman correlations were employed to examine the relationship between the score of risk and the scores of stromal and immune. Two-way ANOVA was used to identify the connections between risk scores and immune infiltration subtypes. Finally, the two-way ANOVA analysis was carried out to identify the connections of risk scores and immune infiltration subtypes. To determine the gene signature’s stem cell-like features, Spearman correlations were conducted to evaluate the connections between tumor stemness and scores of signature.

2.6.Chemotherapy sensitivity analysis

First, 218 chemotherapy drugs (**Supplementary Table 2, Supplementary Digital Content 2, http://links.lww.com/MD/H129**) were retrieved from the CellMiner

database (https://discover.nci.nih.gov/cellminer), after filtering standard FDA certifications and clinical laboratory verifications. Then, Pearson correlation analyses were performed to determine the sensitivity of hub ER stress genes to chemotherapy drugs. The “ggplot2”, “limma”, “ggpubr”, and “impute” R packages were employed to visualize these results.

.

3. Results

3.1.Screening of prognosis-associated DEERGs

The datasets analyzed in the present study are presented in **Figure 1**. A total of 583 ER stress genes were selected from 813 normal and 471 CM tissues, then analyzed to identify DEERGs. Sixty-two ER stress genes were differentially expressed in the TCGA dataset. Among them, eight were connected with the OS of CM patients (**Figure 2A**). The distribution of these genes in normal and tumor samples is shown in **Figure 2B**. Meanwhile, their association with prognosis was verified using univariate Cox analysis (**Figure 2C**). These associations were also observed using correlations (**Figure 2D**). Finally, eight overlapping ER stress genes were identified.

3.2.Construction of a genetic score model for CM patients

Further, the eight DEERGs identified were analyzed using LASSO. Seven ER stress genes were selected to construct the risk signature model: Z-DNA Binding Protein 1 (ZBP1), Diablo IAP-Binding Mitochondrial Protein (DIABLO), Granulysin (GNLY), Fas Ligand (FASLG), Aurora Kinase A (AURKA), TNF Receptor Superfamily Member 21 (TNFRSF21), and CD40 Ligand (CD40LG) (**Table 1, Supplementary Figure 1, Supplementary Digital Content 3,http://links.lww.com/MD/H130**). According to the median risk scores, the patients in TCGA (**Figure 3A and B**) and GEO (**Figure 3C and D**) cohorts were divided into high- and low-risk groups. The different directions distributed in the two groups were found in both TCGA (**Figure 3E and F**) and GEO (**Figure 3G and H**) cohorts during the PCA and t-SNE.

3.3.Correlations between the risk scores and clinical characteristics of CM patients

In the TCGA cohort, reduced OS was observed for high-risk CM patients ($p < 0.001$; **Figure 3I**), further confirmed in the validation cohort ($p < 0.001$; **Figure 3K**). The Receiver Operating Characteristic (ROC) analysis indicated that our risk signature presented a moderate predictive accuracy at one (AUC = 0.642), two (AUC = 0.684), and three (AUC = 0.642) years of follow-up in the TCGA-CM cohort (**Figure 3J**). Compared with the TCGA cohort, the AUC slightly decreased in the validation cohort,

presenting an AUC of 0.619 for one, 0.624 for two, and 0.610 for three years of follow-up (**Figure 3L**). These results confirmed that our signature model could sensitively and specifically predict the OS of CM patients.

Multivariate and univariate Cox regressions showed that, in CM patients, the risk score was an independent prognostic factor, whereas the risk signature was associated with prognosis (**Figure 4A and B**). Moreover, higher risk scores were observed in male CM patients ($p < 0.05$, **Figure 4C**). Meanwhile, a significant correlation between higher T stages and higher risk scores was observed in CM patients ($p < 0.05$, **Figure 4D**). These analyses indicated that our risk signature is connected to CM development.

3.4.Correlations with immune response, tumor stemness, and m6A-related genes

Compared to the low-risk score subgroup, the levels of nearly all related functions, pathways, and the proportion of immune cell subpopulations (except mast cells) were greatly reduced in the high-risk score group, in both TCGA (**Figure 5A and B**) and validation (**Figure 5C and D**) cohorts. Similar results were obtained using EPIC, MCP counter, XCELL, CIBERSORT, QUANTISEQ, and TIMER (**Figure 5C**). Regarding immune checkpoints, all ER stress-related genes identified were more expressed in the low-risk subgroup, except for *CD276* (**Figure 5F**). Moreover, for tumor promotion and suppression, the immune infiltrates, including wound healing (C1), INF-g dominant (C2), inflammatory (C3), and lymphocyte-depleted (C4), were calculated to explore the relationship between the risk signature and immune components [29]. The results showed that the risk scores were significantly higher for C1 and C4 subtypes and lower for C2 (**Figure 5G**). Meanwhile, considering the connection between the ER stress genes identified and immune components, the genes *ZBP1*, *GNLY*, *FASLG*, and *CD40LG* were upregulated (**Supplementary Figure 2A–D,**) and significantly connected with the C2 immune subtype. On the other hand, C1 and C4 subtypes were connected to the downregulation of these genes. Meanwhile, no significant differences were detected between immune subtypes and the risk scores of *DIABLO*, *AURKA*, and *TNFRSF21* genes (**Supplementary Figure 2E–G**).

Other possible regulators of CM progression, such as tumor immune

microenvironment (stromal and immune scores), m6A-related genes, as well as tumor stemness (DNA methylation pattern and RNA stemness score). The gene signature constructed was significantly and negatively associated with the immune microenvironment ($p < 0.05$; **Figure 5H and I**), but positively associated with tumor stemness ($p < 0.05$; **Figure 5J and K**). The correlation between hub ER stress genes and tumor immune microenvironment and stemness (**Supplementary Figure 3**) showed that *ZBP1*, *GNLY*, *FASLG*, *TNFRSF21*, and *CD40LG* were positively connected but *DIABLO* and *AURKA* were negatively associated with the tumor immune microenvironment; meanwhile, *ZBP1*, *FASLG*, *TNFRSF21*, and *CD40LG* were negatively associated but *DIABLO* and *AURKA* were positively related with tumor stemness. Besides, no significant association was observed between *GNLY* and DNAss and RNAss. The m6A-related genes *HNRNPC*, *RBM15*, ZC3H13, *YTHDF1*, *YTHDF2*, and *FTO* were more expressed and *WTAP* was less expressed in the high-risk group in comparison to the low-risk group (**Figure 5L**).

Moreover, considering the roles of the immune checkpoint proteins PD-L1 and PD-L2 in immune progression, we analyzed the correlation between these loci and the gene signature. Both *PD-L1* and *PD-L2* were significantly less expressed in the high-risk group and were negatively related to the CM risk signature (**Figure 6A-D**).

3.5.Functional enrichment analyses

In the GO analysis, predominant enrichment of hub genes was observed in different biological processes (BP), including neuron apoptotic process, positive regulation of endothelial cell apoptotic process, and necroptotic process (**Figure 7A and B**). In the cellular components (CC) category, hub ER stress-related genes were enriched in the CD40 receptor complex, pronucleus, and spindle pole centrosome. Moreover, tumor necrosis factor receptor binding, cytokine activity, and cytokine receptor binding were enriched in the molecular functions (MF) category. Meanwhile, the KEGG enrichment analysis indicated that the genes associated with pyroptosis were enriched in the allograft rejection, autoimmune thyroid disease, cytokine-cytokine receptor interaction, apoptosis, and necroptosis pathways (**Figure 7C and D**). Additionally, the KEGG

enrichment analysis carried out using the GSEA software (**Figure 7E**) revealed that, in the high-risk group, 19 pathways, including pyrimidine metabolism, nucleotide excision repair, and RNA polymerase, were significantly enriched (**Supplementary Table 3**). Meanwhile, in the group with low-risk scores, 46 pathways, including chemokine signaling pathways, Jak stat signaling pathway, and natural killer cell-mediated cytotoxicity, were enriched. Altogether, these results indicated possible underlying mechanisms of ER stress genes in CM.

3.6.Prognostic value of the hub genes selected

In the high-risk group, significantly elevated gene expressions were found for the ER stress genes *DIABLO* and *AURKA* compared to the low-risk group. Meanwhile, ZBP1, GNLY, FASLG, TNFRSF21, and CD40LG were less expressed (**Figure 8A–G**). Then, the correlation analyses showed that the expression of *DIABLO* and *AURKA* was positively associated, and ZBP1, GNLY, FASLG, TNFRSF21, and CD40LG were negatively associated with risk scores (**Figure 8H-N**). Considering the correlation of ER stress genes and CM tissues, abundant expression of ZBP1, GNLY, FASLG, AURKA, TNFRSF21, and CD40LG, and less *DIABLO* expression were observed in CM samples than in healthy controls (**Figure 8O–U**). Finally, the Kaplan-Meier survival analysis was used to examine the prognostic value of ER stress genes. the expression of *DIABLO* and *AURKA* was negatively associated with the prognosis of CM patients, while ZBP1, GNLY, FASLG, TNFRSF21, and CD40LG were positively associated with survival (**Figure 8V–AB**).

3.7.Connections between hub ER stress genes and drug sensitivity

Hub ER stress genes were sensitively and respectively correlated to chemotherapy drugs ($p < 0.05$; **Supplementary Table 4**). For example, the increased sensitivity to LDK-378 and alectinib was positively correlated with increased ZBP1 expression (**Figure 9**). In contrast, increased TNFRSF21 expression was negatively associated with the sensitivity to etoposide, teniposide, melphalan, ifosfamide, tfdu, nitrogen mustard, valrubicin, uracil mustard, lomustine, epirubicin, and triethylenemelamine.

4. Discussion

Although increasing melanoma biomarkers have been identified using next-generation sequencing, novel markers associated with early prognosis and more close detection are still needed for CM patients. The ER stress is significantly correlated with skin cells differentiation, and can also participate in melanoma progression. Meanwhile, the role and function of ER stress in CM have not been studied systemically. Moreover, no ER stress-related gene signature has been identified yet. Similar to previously established risk signatures, such as immune checkpoint- [30], ferroptosis- [31], and hypoxia-related signatures [32], our current risk gene signature displayed high predictive accuracy for the prognosis of CM patients. This gene signature was also connected with the TME, immune components, immune status, tumor stemness, m6A-related genes, and susceptibility to chemotherapeutic drugs, thereby presenting an advantage over other gene signatures.

In the present study, 583 ER stress genes were systematically analyzed to identify those associated with the OS of CM patients. Next, seven hub genes (ZBP1, DIABLO, GNLY, FASLG, AURKA, TNFRSF21, and CD40LG) were used to construct a novel prognostic signature for CM. The survival and ROC analyses indicated that the gene signature was not only significantly connected with the OS of CM patients but also showed high accuracy for prognosis prediction. The signature was also correlated with the T stage of CM, revealing the effectiveness of this gene signature for predicting the tumor growth of CM.

Based on the GSEA, the risk signature was enriched in several immune-related pathways, including the T cell receptor signaling pathway, the Toll-like receptor signaling pathway, as well as natural killer cell-mediated cytotoxicity. Thus, the prognostic value of the gene signature might be attributed to its association with immune processes. Nearly all immune cells, except mast cells, presented reduced infiltration and immune functions in the high-risk score group. Since these infiltrated immune cells play important roles in the stimulation of anti-tumor immunity [33], the

degree of anti-tumor immunity of SKCM patients in the high-risk group was substantially reduced. Additionally, negative correlations between both of the immune and stromal cell scores and risk scores were observed from the ESTIMATE algorithm analysis, suggesting the inhibited infiltration of immune cells in the high-risk group. Moreover, regarding the correlation of CM and immune components, we found that C2 was significantly associated with the risk scores. Considering the predictive value of the gene signature in CM prognosis, C2 might also be a protective factor in CM.

Cancer immunotherapies targeting immune checkpoints have improved the outcomes of various cancers [34]. However, they have different effects depending on the tumor type. Both PD-L1 and PD-L2 are virtual regulators of immune responses [35]. Additionally, some tumors express immune-inhibitory checkpoint cytokines, contributing to the suppression of immune responses mediated by T cells. The binding of PD1 on T cells and its ligand PD-L1 in tumor cells can induce the immune escape of tumor cells and exhaustion of T cells [36]. However, better clinical outcomes were also positively correlated with the expression level of PD-L1 on melanoma cells. Through blocking the PD1/PD-L1 binding-mediated inhibition and enhancing the function of T cells, impressive outcomes were observed after treatment with monoclonal antibodies targeting the PD-1/PD-L1 pathway in clinical trials [37,38]. The significantly differential expression of PD-L1 and PD-L2 in our gene risk groups, as well as the fact that they are both negatively correlated with the risk scores, were also verified in this study. The levels of nearly all immune checkpoints were significantly lower in the high-risk group, suggesting that immune responses were greatly altered in this group. Overall, our prognostic gene signature could predict the expression of immune checkpoints in CM and potentially guide immunotherapy implementation. However, the specific relationship between ER stress genes and immune genes requires further study.

Cancer stem cell-like cells (CSCs) promote cancer progression due to their invasion and self-renewal abilities [39,40]. In the present study, the ER stress gene signature was positively connected with stem cell score, confirming that this signature

was a risk factor for CM. The m6A-related genes comprehend another tumor research hotspot [41]. Our ER stress gene signature could effectively predict the expression levels of m6A-related genes in CM, including HNRNPC, RBM15, ZC3H13, WTAP, YTHDF1, FTO, and YTHDF2. However, the specific mechanisms underlying these relationships need further exploration.

Despite the prognostic value of the current risk signature, this study also has some limitations. First, the results from our present retrospective study need further confirmation by prospective studies. Second, more experimental assays are needed to verify and validate the conclusions obtained from bioinformatics analyses. In the future, functional studies should be performed to gain mechanistic insights into ER stress genes and their role in CM development.

5. Conclusions

In the present study, a novel prognostic risk signature consisting of seven hub ER stress-related genes was constructed and presented high predictive accuracy. This gene signature was valuable to predict parameters related to immune components, immune functions, immune cell infiltration, tumor microenvironment, stemness, m6A-related genes, and drug sensitivity in CM patients. To the best of our knowledge, this is the first ER stress-associated gene signature for CM. These results also provided a novel basis for understanding the specific effects of ER stress genes in CM. Therefore, this study comprehends a significant contribution to the literature and can contribute to improvements in the outcomes and individualized treatments for CM patients

Author contributions

Conceptualization: Rong Chen.

Data curation: Rong Chen.

Formal analysis: Rong Chen.

Methodology: Linjun Niu.

Project administration: Kangjie Hong.

Writing – original draft: Liang Wu and Youwu He.

Writing – review & editing: Gang Liu

Acknowledgements

Not applicable.

Funding

Not applicable.

Availability of data and materials

The datasets analysed during the current study are available sourced from the publicly available TCGA database (https://portal.gdc.cancer.gov) and GEO database (https://www.ncbi.nlm.nih.gov/geo/).

Ethics approval and consent to participate

Not applicable.

Patient consent for publication

Not applicable.

Competing interests

The authors declare that they have no competing interests.

References

1. Ekwueme DU, Guy GP, Jr., Li C, Rim SH, Parelkar P, Chen SC. The health burden and economic costs of cutaneous melanoma mortality by race/ethnicity-United States, 2000 to 2006. *J Am Acad Dermatol*. Nov 2011;65(5 Suppl 1):S133-43. doi:10.1016/j.jaad.2011.04.036

2. Kanavy HE, Gerstenblith MR. Ultraviolet radiation and melanoma. *Semin Cutan Med Surg*. Dec 2011;30(4):222-8. doi:10.1016/j.sder.2011.08.003

3. Pawlikowska M, Piotrowski J, Jędrzejewski T, Kozak W, Slominski AT, Brożyna AA. Coriolus versicolor-derived protein-bound polysaccharides trigger the caspase-independent cell death pathway in amelanotic but not melanotic melanoma cells. *Phytother Res*. Jan 2020;34(1):173-183. doi:10.1002/ptr.6513

4. Slominski A, Kim TK, Brożyna AA, et al. The role of melanogenesis in regulation of melanoma behavior: melanogenesis leads to stimulation of HIF-1α expression and HIF-dependent attendant pathways. *Arch Biochem Biophys*. Dec 1 2014;563:79-93. doi:10.1016/j.abb.2014.06.030

5. Brożyna AA, Jóźwicki W, Carlson JA, Slominski AT. Melanogenesis affects overall and disease-free survival in patients with stage III and IV melanoma. *Hum Pathol*. Oct 2013;44(10):2071-4. doi:10.1016/j.humpath.2013.02.022

6. Slominski A, Zmijewski MA, Pawelek J. L-tyrosine and L-dihydroxyphenylalanine as hormone-like regulators of melanocyte functions. *Pigment Cell Melanoma Res*. Jan 2012;25(1):14-27. doi:10.1111/j.1755-148X.2011.00898.x

7. Scisłowski PW, Słomiński A, Bomirski A. Biochemical characterization of three hamster melanoma variants--II. Glycolysis and oxygen consumption. *Int J Biochem*. 1984;16(3):327-31. doi:10.1016/0020-711x(84)90107-1

8. Slominski A, Zbytek B, Slominski R. Inhibitors of melanogenesis increase toxicity of cyclophosphamide and lymphocytes against melanoma cells. *Int J Cancer*. Mar 15 2009;124(6):1470-7. doi:10.1002/ijc.24005

9. Slominski A, Friedrich T. L-dopa inhibits in vitro phosphorylation of melanoma glycoproteins. *Pigment Cell Res*. Dec 1992;5(6):396-9. doi:10.1111/j.1600-0749.1992.tb00569.x

10. Li W, Slominski R, Slominski AT. High-resolution magic angle spinning nuclear magnetic resonance analysis of metabolic changes in melanoma cells after induction of melanogenesis. *Anal Biochem*. Mar 15 2009;386(2):282-4. doi:10.1016/j.ab.2008.12.017

11. Bray F, Ferlay J, Soerjomataram I, Siegel RL, Torre LA, Jemal A. Global cancer statistics 2018: GLOBOCAN estimates of incidence and mortality worldwide for 36 cancers in 185 countries. *CA Cancer J Clin*. Nov 2018;68(6):394-424. doi:10.3322/caac.21492

12. Finn L, Markovic SN, Joseph RW. Therapy for metastatic melanoma: the past, present, and future. *BMC Med*. Mar 2 2012;10:23. doi:10.1186/1741-7015-10-23

13. Gershenwald JE, Scolyer RA, Hess KR, et al. Melanoma staging: Evidence-based changes in the American Joint Committee on Cancer eighth edition cancer staging

manual. *CA Cancer J Clin*. Nov 2017;67(6):472-492. doi:10.3322/caac.21409
14. Gilchrest BA, Eller MS, Geller AC, Yaar M. The pathogenesis of melanoma induced by ultraviolet radiation. *N Engl J Med*. Apr 29 1999;340(17):1341-8. doi:10.1056/nejm199904293401707
15. Hawkes JE, Truong A, Meyer LJ. Genetic predisposition to melanoma. *Semin Oncol*. Oct 2016;43(5):591-597. doi:10.1053/j.seminoncol.2016.08.003
16. Jain BP. An Overview of Unfolded Protein Response Signaling and Its Role in Cancer. *Cancer Biother Radiopharm*. Oct 2017;32(8):275-281. doi:10.1089/cbr.2017.2309
17. Urra H, Dufey E, Avril T, Chevet E, Hetz C. Endoplasmic Reticulum Stress and the Hallmarks of Cancer. *Trends Cancer*. May 2016;2(5):252-262. doi:10.1016/j.trecan.2016.03.007
18. Sugiura K, Muro Y, Futamura K, et al. The unfolded protein response is activated in differentiating epidermal keratinocytes. *J Invest Dermatol*. Sep 2009;129(9):2126-35. doi:10.1038/jid.2009.51
19. Park K, Lee SE, Shin KO, Uchida Y. Insights into the role of endoplasmic reticulum stress in skin function and associated diseases. *Febs j*. Jan 2019;286(2):413-425. doi:10.1111/febs.14739
20. Shimizu A, Kaira K, Yasuda M, Asao T, Ishikawa O. Clinical and Pathological Significance of ER Stress Marker (BiP/GRP78 and PERK) Expression in Malignant Melanoma. *Pathol Oncol Res*. Jan 2017;23(1):111-116. doi:10.1007/s12253-016-0099-9
21. Ma XH, Piao SF, Dey S, et al. Targeting ER stress-induced autophagy overcomes BRAF inhibitor resistance in melanoma. *J Clin Invest*. Mar 2014;124(3):1406-17. doi:10.1172/jci70454
22. Alasiri G, Fan LY, Zona S, et al. ER stress and cancer: The FOXO forkhead transcription factor link. *Mol Cell Endocrinol*. Feb 15 2018;462(Pt B):67-81. doi:10.1016/j.mce.2017.05.027
23. Wang M, Law ME, Castellano RK, Law BK. The unfolded protein response as a target for anticancer therapeutics. *Crit Rev Oncol Hematol*. Jul 2018;127:66-79. doi:10.1016/j.critrevonc.2018.05.003
24. Xiao Y, Zhu Z, Li J, et al. Expression and prognostic value of long non-coding RNA H19 in glioma via integrated bioinformatics analyses. *Aging (Albany NY)*. Feb 20 2020;12(4):3407-3430. doi:10.18632/aging.102819
25. Zhang M, Wang X, Chen X, Zhang Q, Hong J. Novel Immune-Related Gene Signature for Risk Stratification and Prognosis of Survival in Lower-Grade Glioma. *Front Genet*. 2020;11:363. doi:10.3389/fgene.2020.00363
26. Bai J, Zhang X, Xiang ZX, Zhong PY, Xiong B. Identification of prognostic immune-related signature predicting the overall survival for colorectal cancer. *Eur Rev Med Pharmacol Sci*. Feb 2020;24(3):1134-1141. doi:10.26355/eurrev_202002_20164
27. Huang da W, Sherman BT, Lempicki RA. Systematic and integrative analysis of large gene lists using DAVID bioinformatics resources. *Nat Protoc*. 2009;4(1):44-57.

doi:10.1038/nprot.2008.211
28. Yoshihara K, Shahmoradgoli M, Martínez E, et al. Inferring tumour purity and stromal and immune cell admixture from expression data. *Nat Commun*. 2013;4:2612. doi:10.1038/ncomms3612
29. Tamborero D, Rubio-Perez C, Muiños F, et al. A Pan-cancer Landscape of Interactions between Solid Tumors and Infiltrating Immune Cell Populations. *Clin Cancer Res*. Aug 1 2018;24(15):3717-3728. doi:10.1158/1078-0432.Ccr-17-3509
30. Tian M, Yang J, Han J, He J, Liao W. A novel immune checkpoint-related seven-gene signature for predicting prognosis and immunotherapy response in melanoma. *Int Immunopharmacol*. Oct 2020;87:106821. doi:10.1016/j.intimp.2020.106821
31. Luo H, Ma C. A Novel Ferroptosis-Associated Gene Signature to Predict Prognosis in Patients with Uveal Melanoma. *Diagnostics (Basel)*. Feb 2 2021;11(2)doi:10.3390/diagnostics11020219
32. Shou Y, Yang L, Yang Y, Zhu X, Li F, Xu J. Identification of Signatures of Prognosis Prediction for Melanoma Using a Hypoxia Score. *Front Genet*. 2020;11:570530. doi:10.3389/fgene.2020.570530
33. Shankaran V, Ikeda H, Bruce AT, et al. IFNgamma and lymphocytes prevent primary tumour development and shape tumour immunogenicity. *Nature*. Apr 26 2001;410(6832):1107-11. doi:10.1038/35074122
34. Chinai JM, Janakiram M, Chen F, Chen W, Kaplan M, Zang X. New immunotherapies targeting the PD-1 pathway. *Trends Pharmacol Sci*. Sep 2015;36(9):587-95. doi:10.1016/j.tips.2015.06.005
35. Lin Z, Xu Q, Miao D, Yu F. An Inflammatory Response-Related Gene Signature Can Impact the Immune Status and Predict the Prognosis of Hepatocellular Carcinoma. *Front Oncol*. 2021;11:644416. doi:10.3389/fonc.2021.644416
36. Wang X, Teng F, Kong L, Yu J. PD-L1 expression in human cancers and its association with clinical outcomes. *Onco Targets Ther*. 2016;9:5023-39. doi:10.2147/ott.S105862
37. Allison JP. Immune Checkpoint Blockade in Cancer Therapy: The 2015 Lasker-DeBakey Clinical Medical Research Award. *Jama*. Sep 15 2015;314(11):1113-4. doi:10.1001/jama.2015.11929
38. Ohaegbulam KC, Assal A, Lazar-Molnar E, Yao Y, Zang X. Human cancer immunotherapy with antibodies to the PD-1 and PD-L1 pathway. *Trends Mol Med*. Jan 2015;21(1):24-33. doi:10.1016/j.molmed.2014.10.009
39. Huang Z, Cheng L, Guryanova OA, Wu Q, Bao S. Cancer stem cells in glioblastoma--molecular signaling and therapeutic targeting. *Protein Cell*. Jul 2010;1(7):638-55. doi:10.1007/s13238-010-0078-y
40. Schonberg DL, Lubelski D, Miller TE, Rich JN. Brain tumor stem cells: Molecular characteristics and their impact on therapy. *Mol Aspects Med*. Oct 2014;39:82-101. doi:10.1016/j.mam.2013.06.004
41. Liao Y, Han P, Zhang Y, Ni B. Physio-pathological effects of m6A modification and its potential contribution to melanoma. *Clin Transl Oncol*. Nov 2021;23(11):2269-

2279. doi:10.1007/s12094-021-02644-3

Figure legends

Figure 1. Schema of the study.

Figure 2. Identification of candidate prognostic DEGs in TCGA-CM cohort. (A) Venn diagram of ER-related genes determined by differential expression and univariate Cox analyses. (B) Heatmap of candidate prognostic DEGs in TCGA-CM cohort. (C) Forest plots of correlations between candidate prognostic DEGs and overall survival of patients in TCGA-CM cohort. (D) Correlation network of candidate prognostic DEGs.

Figure 3. Prognostic analysis of risk signature. Risk score distribution (A, C) and survival status (B, D) of TCGA-CM and GEO cohorts, respectively. PCA plot (E) and t-SNE (F) analysis of TCGA-CM cohort. PCA plot (G) and t-SNE (H) analysis of validation cohort. (I) Survival curve of the TCGA cohort. (J) TimeROC curves to forecast overall survival of patients from TCGA-CM cohort. (K) Survival curve of validation cohort. (L) TimeROC curves to forecast overall survival of patients in validation cohort.

Figure 4. Associations between risk signature and clinicopathological factors. Univariate (A) and multivariate Cox (B) regression of clinicopathological features in TCGA-CM cohort. Correlations between risk scores and gender (C) and T stage (D) in TCGA-CM cohort.

Figure 5. Potential role of risk signature in CM immune status, tumor stemness, and m6A-related genes. Boxplots of scores of immune cells (A) and immune-associated functions (B) in risk subgroups of TCGA-CM cohort. Boxplots of scores for immune cells (C) and immune-associated functions (D) in risk subgroups of validation cohort. (E) Heatmap for immune responses based on EPIC, XCELL, MCP counter, QUANTISEQ, CIBERSORT, and TIMER among two risk subgroups. Associations between risk signature and immune checkpoints (F), immune infiltration subtypes (G), stromal scores (H), immune scores (I), RNAss (J), DNAss (K), and m6A-related genes (L).

Figure 6. Associations between risk signature and immune checkpoints. Expression levels of genes PD-L1 (A) and PD-L2 (C) in risk subgroups. Correlation analysis between risk score, PD-L1 (B), and PD-L2 (D).

Figure 7. Functional enrichment analysis. (A, B) GO enrichment terms of hub ER genes in CC, BP, and MF. (C, D) KEGG enrichment terms of hub ER genes. (E) Gene Set Enrichment Analysis of top 10 enriched pathways in risk signature.

Figure 8. Roles of ER stress genes in risk signature and CM prognosis. Expression of *ZBP1* (A), *DIABLO* (B), *GNLY* (C), *FASLG* (D), *AURKA* (E), *TNFRSF21* (F), and *CD40LG* (G) genes in risk subgroups. Correlations between risk signature and *ZBP1* (H), *DIABLO* (I), *GNLY* (J), *FASLG* (K), *AURKA* (L), *TNFRSF21* (M), and *CD40LG* (N) genes. Expression of *ZBP1* (O), *DIABLO* (P), *GNLY* (Q), *FASLG* (R), *AURKA* (S), *TNFRSF21* (T), and *CD40LG* (U) genes in CM. Kaplan-Meier curves of TCGA-CM cohort verify prognostic value of *ZBP1* (V), *DIABLO* (W), *GNLY* (X), *FASLG* (Y), *AURKA* (Z), *TNFRSF21* (AA), and *CD40LG* (AB).

Figure 9. Scatter plots of top 16 classes of associations between ER stress genes and drug sensitivity.

Supplementary Figure 1. LASSO findings of factors and construction of risk signature. (A) LASSO coefficient profiles of candidate prognostic DEGs. (B) Selection of penalty parameter (λ) in LASSO model.

Supplementary Figure 2. Expression of ER genes *ZBP1* (A), *GNLY* (B), *FASLG* (C), *CD40LG* (D), *DIABLO* (E), *AURKA* (F), *TNFRSF21* (G), and in different immune infiltrate subtypes.

Supplementary Figure 3. Potential roles of genes in CM immune status and tumor stemness. Associations between *ZBP1* (A), *DIABLO* (C), *GNLY* (E), *FASLG* (G), *AURKA* (I), *TNFRSF21* (K), and *CD40LG* (M) genes and stromal and immune scores, respectively. Associations between *ZBP1* (B), *DIABLO* (D), *GNLY* (F), *FASLG* (H), *AURKA* (J), *TNFRSF21* (L), and *CD40LG* (N) genes and DNAss and RNAss, respectively.

Supplementary Table 1. The protein domains of ER stress genes from the GeneCards database.

Supplementary Table 2. The chemotherapy drugs retrieved from the CellMiner database.

Supplementary Table 3. The KEGG enrichment analysis carried out using the GSEA software.

Supplementary Table 4. Connections between hub ER stress genes and chemotherapy drugs.
